# Identification and phylogenetic analysis of voltage-gated sodium channel haplotypes in the malaria vector *Anopheles sinensis* using a high-throughput amplicon sequencing approach

**DOI:** 10.1186/s13071-021-05009-5

**Published:** 2021-09-26

**Authors:** Ruoyao Ni, Nian Liu, Mei Li, Weiping Qian, Xinghui Qiu

**Affiliations:** 1 State Key Laboratory of Integrated Management of Pest Insects and Rodents, Institute of Zoology, Chinese Academy of Sciences, Beijing, 100101 China; 2 Sichuan Center for Disease Control and Prevention, Chengdu, China; 3 University of Chinese Academy of Sciences, Beijing, China

**Keywords:** *Anopheles sinensis*, Next-generation sequencing, Voltage-gated sodium channel, Knock-down resistance, Haplotype, Phylogenetic analysis

## Abstract

**Background:**

*Anopheles sinensis* is a dominant vector for malaria transmission in Asian countries. Voltage-gated sodium channel (VGSC) mutation-mediated knock-down resistance (*kdr*) has developed in many *A. sinensis* populations because of intensive and long-term use of pyrethroids. Our previous study showed that multiple mutations at position 1014 of the VGSC were heterogeneously distributed in *A. sinensis* populations across Sichuan, China.

**Methods:**

To understand resistance genotypes at the haplotype level and reconstruct the phylogenetic relationship of *VGSC* haplotypes, a cost-effective next-generation sequencing (NGS)-based amplicon sequencing approach was established to clarify haplotypes containing codon 1014 of the *VGSC* gene from a total of 446 adults collected in 12 locations of Sichuan, China.

**Results:**

Nineteen (19) haplotypes were identified, including 11 wild 1014L, 6 resistance 1014F, and 2 resistance 1014C haplotypes. We found that resistance haplotypes of *A. sinensis VGSC* were widely distributed at frequencies ranging from 3.67 to 92.61%. The frequencies of the 1014C haplotype in the southeast of Sichuan (Luzhou, Guangan, and Suining) were relatively higher than those in other sampling locations. Phylogenetic analyses support that *kdr*-type mutation at position 1014 is not singly originated and resistance 1014C haplotypes evolve from TTT-encoding 1014F.

**Conclusions:**

A cost-effective next-generation sequencing (NGS)-based amplicon sequencing approach has been established in this study. The data revealed the patchy distribution of *VGSC* resistance haplotypes with overall high frequencies in Sichuan, China. Phylogenetic analyses support multiple origins and sequential evolution (1014L → 1014F → 1014C) for *kdr*-type mutations in *A. sinensis*.

**Graphical abstract:**

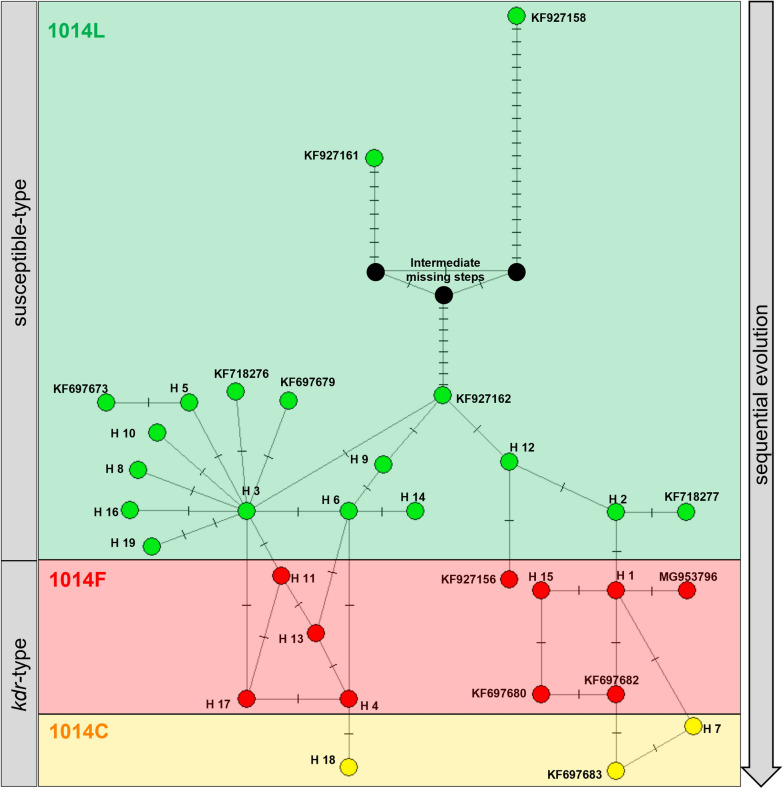

## Background

Chemical insecticides have been the primary weapon for preventing and controlling vector-borne diseases, including malaria, in the past decades [1]. The World Health Organization (WHO)-recommended pyrethroids are the most commonly used insecticides in China for indoor residual spray (IRS), insecticide-treated bed nets (ITNs), and mosquito coils [2]. Many studies have demonstrated that mutations at position 1014 of the VGSC (numbered after the *Musca domestica* para-type sodium channel protein, GenBank: CAA65448.1), such as 1014F/H, can reduce the sensitivity of arthropod VGSCs to pyrethroids and cause so-called knock-down resistance (*kdr*) [3–9].

**Fig. 1:**
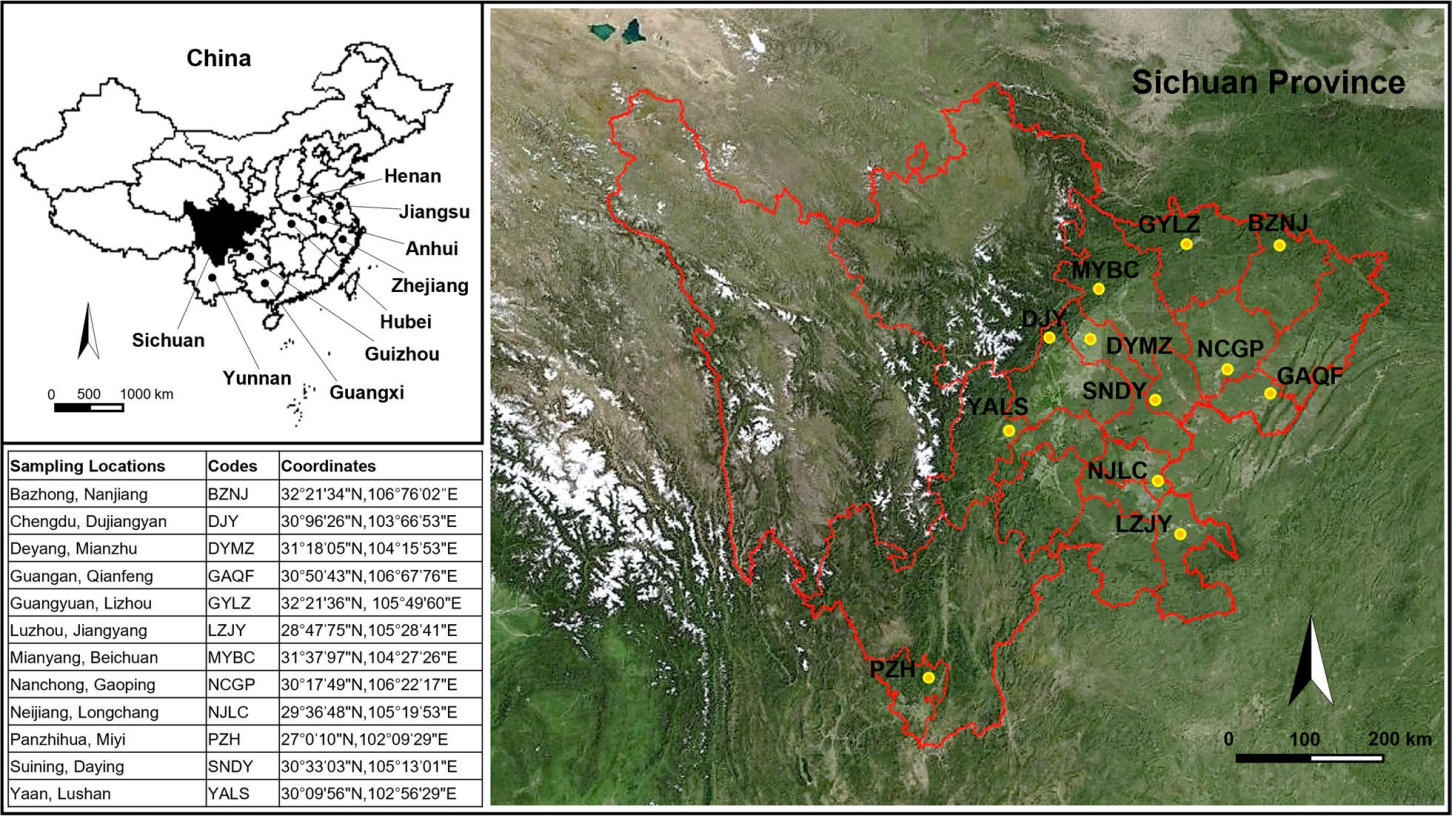
Geographical information about mosquito sampling locations

Sichuan province is located in the southwest of China (Fig. 1). The natural environment in most parts of Sichuan is suitable for the breeding of malaria vectors. Historically, Sichuan has been a malaria-endemic region in China. Although no indigenous malaria case has been reported since 2011, the increasing imported cases indicate a potential risk for the re-emergence of malaria in Sichuan [10, 11]. The intensive use of insecticides such as deltamethrin and permethrin for mosquito-targeted control and in agriculture has led to the development of insecticide resistance in the primary malaria vector *Anopheles sinensis* in this region [12, 13].

A recent study detected multiple mutations at position 1014 of the VGSC in Sichuan *A. sinensis* by Sanger sequencing [14]. However, due to the diverse polymorphisms in the *VGSC* gene, direct DNA sequencing of PCR product from heterozygous individuals is incapable of haplotype identification. The identification of haplotype is useful for understanding the origin and evolution of *kdr*-associated mutations and helpful for monitoring distribution and spread of *kdr* haplotypes. In this context, we adopted the NGS platform to establish an amplicon-based sequencing pipeline to clarify *VGSC* haplotypes. The specific objectives of this effort were (i) to identify *VGSC* haplotypes and delineate their geographical distribution in *A. sinensis* populations across Sichuan and (ii) to elucidate the evolutionary relationship of *kdr*-type resistance mutations.

## Methods

### *Anopheles sinensis* collections

A total of 446 adult *A. sinensis* were caught from 12 sampling locations across Sichuan from August to September in 2018. The sampling locations are shown in Fig. 1.

### *Anopheles sinensis* genomic DNA (gDNA) preparation

**Fig. 2:**
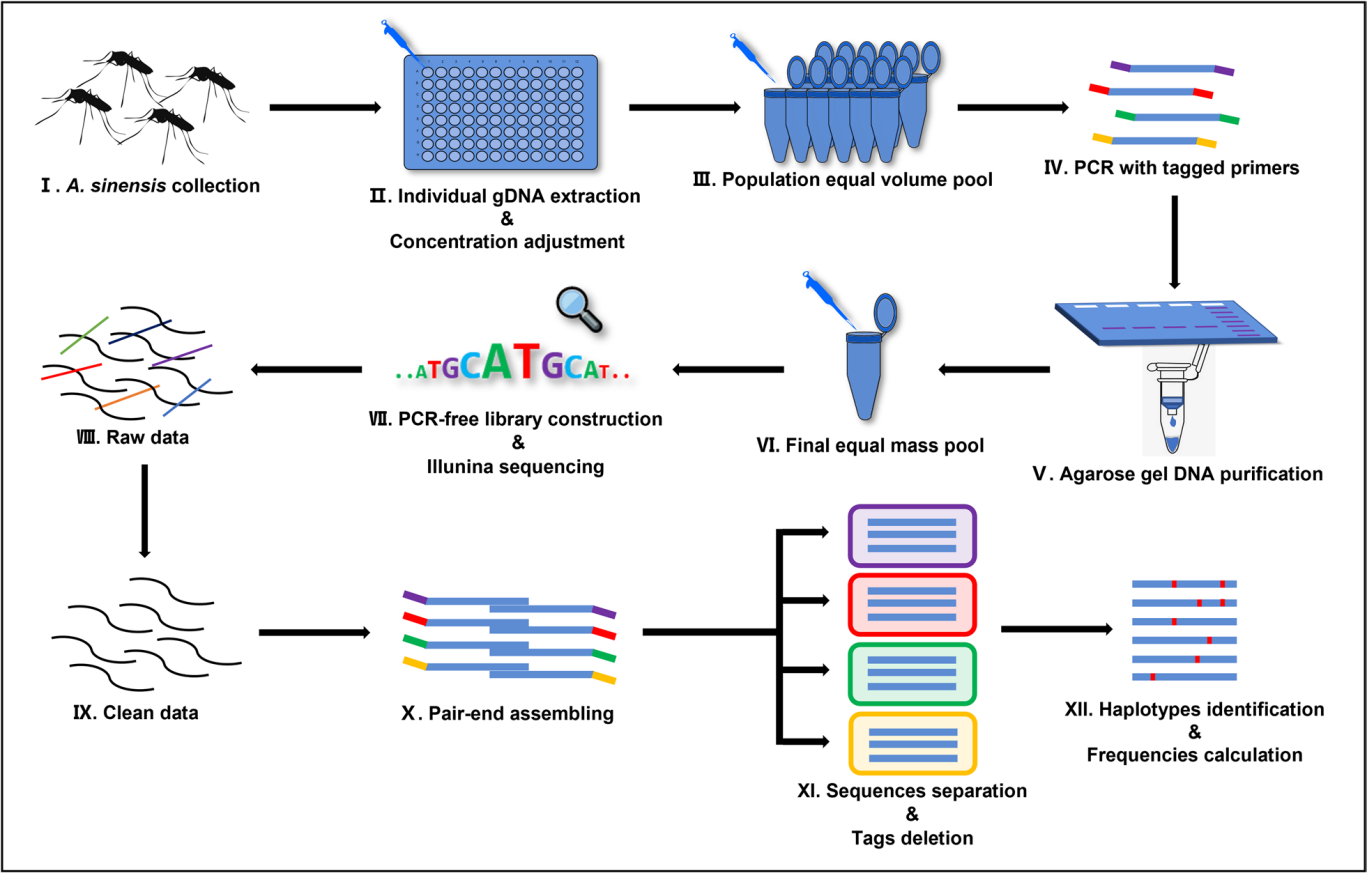
Pipeline of NGS-based *VGSC* amplicon sequencing

The gDNA was isolated from each mosquito using the protocol of Rinkevinch et al. [15]. The gDNA concentration for each mosquito was detected by Nanodrop 2000 (Thermo, USA) and then adjusted to 50 ng/μl with ddH_2_O. Then, the gDNA of individual mosquitoes from the same sampling location was pooled in equal volume (10 μl). In this manner, 12 pooled gDNA samples representing 12 populations were obtained and stored at – 20 ℃ until subsequent treatments. The procedure is shown schematically in Fig. 2 (I–III).

### *VGSC* amplicon preparation and sequencing

Primers with population-specific tags for amplicon preparation were designed based on the primers used to amplify a 325-bp genomic region that contains codon 1014 and a complete intron of the *VGSC* (*kdr-*F: 5′-TGCCACTCCGTGTGTTTAGA; *kdr-*R: 5′-GAGCGATGATGATCCGAAAT) [16]. The population-specific tagged sequence (4–6 bp) was added at the 5′ end of the primer pairs. Details of the primers with tag sequences are shown in Table 1. The PCR mixture (50 μl) consisted of 1.5 μl gDNA template, 1 μl *kdr-*F, 1 μl *kdr*-R, 25 μl high-fidelity DNA polymerase (PrimeSTAR® max, Takara, China), and 21.5 μl ddH_2_O. The reaction program was 95 ℃ for 3 min, followed by 30 cycles each with 95 ℃ for 30 s, 55 ℃ for 30 s, 72 ℃ for 30 s, and a final extension of 5 min at 72 ℃. PCR products from each pooled gDNA sample were purified by agarose gel DNA purification kit (Accurate Biology, China) and then pooled with equal mass. DNA libraries were prepared using TruSeq DNA PCR-free kits (Illumina, San Diego, CA, USA). DNA sequencing was performed using the Hiseq2500 sequencer (Illumina, San Diego, CA, USA) with PE250 read type by Novogen Co. Ltd (Beijing, China). The procedure is shown schematically in Fig. 2 (IV–VII).

**Table 1:** Primer sequence information for PCR

Sampling locations	Tag sequences	Tagged *kdr-*F primers	Tagged *kdr-*R primers
BZNJ	ATCACG	ATCACGTGCCACTCCGTGTGTTTAGA	ATCACGGAGCGATGATGATCCGAAAT
DJY	CGATGT	CGATGTTGCCACTCCGTGTGTTTAGA	CGATGTGAGCGATGATGATCCGAAAT
DYMZ	TTAGGC	TTAGGCTGCCACTCCGTGTGTTTAGA	TTAGGCGAGCGATGATGATCCGAAAT
GAQF	TGAC	TGACTGCCACTCCGTGTGTTTAGA	TGACGAGCGATGATGATCCGAAAT
LZJY	ACAGTG	ACAGTGTGCCACTCCGTGTGTTTAGA	ACAGTGGAGCGATGATGATCCGAAAT
GYLZ	CGGT	CGGTTGCCACTCCGTGTGTTTAGA	CGGTGAGCGATGATGATCCGAAAT
MYBC	CAGATC	CAGATCTGCCACTCCGTGTGTTTAGA	CAGATCGAGCGATGATGATCCGAAAT
NCGP	ACTTGA	ACTTGATGCCACTCCGTGTGTTTAGA	ACTTGAGAGCGATGATGATCCGAAAT
NJLC	CACGAT	CACGATTGCCACTCCGTGTGTTTAGA	CACGATGAGCGATGATGATCCGAAAT
PZH	CAACTA	CAACTATGCCACTCCGTGTGTTTAGA	CAACTAGAGCGATGATGATCCGAAAT
SNDY	ACTT	ACTTTGCCACTCCGTGTGTTTAGA	ACTTGAGCGATGATGATCCGAAAT
YALS	ACTGAT	ACTGATTGCCACTCCGTGTGTTTAGA	ACTGATGAGCGATGATGATCCGAAAT

The population-specific tag is underlined

### Bioinformatics pipeline

Two FASTQ format files with a total of 1.496 G raw data were obtained after sequencing. Low-quality sequences were filtered using Trimmomatic [17], and a total of 1.487 G clean data was obtained. Clean data (FASTQ format) were then submitted to pandaseq 2.11 [18] for pair-end assembling. Subsequently, based on population-specific tagged-primer sequences, assembled sequences belonging to the respective populations were separated by the Linux opera system's *grep* command (Ubuntu 20.04 LTS). Sequence reverse complement was performed by Seqtk (https://github.com/lh3/seqtk). For chimera sequence filtering, sequences with both 5′-end and 3′-end matched population-specific tagged-primer were passed. The deletion of pair-end tag sequences was performed by the Linux opera system's *sed* command. After that, identical sequences from each population were merged and counted using FASTX-Toolkit (http://hannonlab.cshl.edu/fastx_toolkit/). The haplotype with a count of less than the threshold was removed (threshold = S/2 N; S is the sum of the number of clean sequences in each population, and N is the sample size from each sampling location). The brief pipeline is presented in Fig. 2 (VIII–XII).

### Data analysis

Referring to the method of Délye et al. [19], Pearson’s correlation analysis was used for assessing the agreement in allele frequencies obtained by Sanger sequencing [14] and the NGS approaches. The Pearson’s correlation coefficient (*r*) was calculated by function *cor ()* in the R programming language. The haplotypes identified in this study and those retrieved from GenBank [20–23] were used for constructing a phylogenetic tree by the maximum likelihood (ML) method and Kimura two-parameter model [24] (1000 bootstrap replicates) in MEGA-X software [25]. Homologous sequences of *Anopheles culicifacies* were used as outgroup. The genealogical network analysis of haplotypes was performed by Network 5.0 (https://www.fluxus-engineering.com/sharenet.htm) using median joining for network calculation [26].

## Results

### Identification of *VGSC* haplotypes

**Fig. 3:**
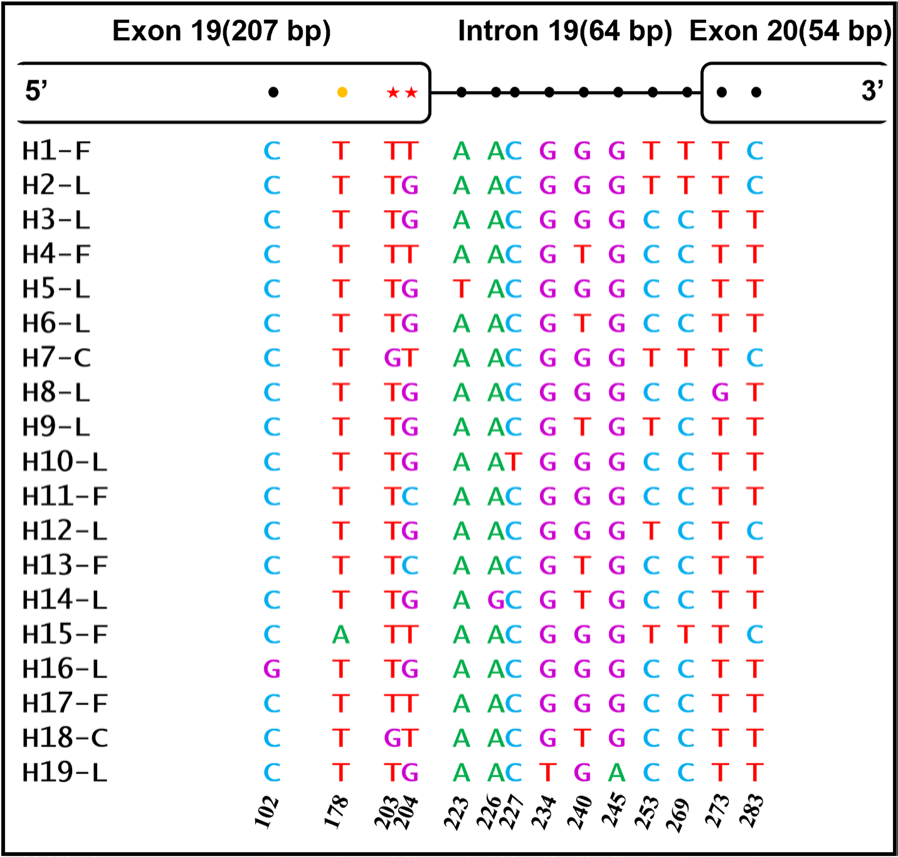
The single nucleotide polymorphic sites identified in this study. Black dots are synonymous mutations in exons or nucleotide variation in introns. The yellow dot indicates non-synonymous mutation leading to conservative amino acid change. Red pentagons represent mutation leading to amino acid substitution at position 1014. H1-F to H19-L are the 19 different haplotypes identified in this study

Fourteen nucleotide polymorphic sites (eight in intron and six in exon) were observed in the amplified fragments (325 bp) of the *A. sinensis VGSC* gene (Fig. 3). The mutations at nucleotides 203 and 204 led to non-conservative amino-acid residue substitutions (L to F or L to C) at position 1014. In addition, a previously reported mutation at locus 178 (A/T) [20, 22], which causes a conservative substitution (L1006I), was also detected. Based on all the DNA sequences, a total of 19 haplotypes were identified, including 11 wild-type 1014L, 6 *kdr*-type 1014F, and 2 *kdr-*type 1014C. Five haplotypes (H8-L, H14-L, H19-L, H11-F, and H18-C) were newly identified in this study (Table 2).

**Table 2:** Distribution of haplotypes identified in this study in China

Haplotype	GenBank accession	Known distribution in China (provinces)
H1-F	MG953791, KF697674, KP763798, KP763776, KP763726	Zhejiang, Anhui, Guangxi, Guizhou, Henan, Hubei, Jiangsu, Shandong, Sichuan
H2-L	MG953793, KF927164, KF718271, KF697669, KP763810, KP763805	Zhejiang, Fujian, Guangxi, Guangdong, Guizhou, Hainan, Henan, Yunnan, Sichuan
H3-L	MG953794, KF927163, KF718272, KF697678, KF697670, KP763809, KP763748	Zhejiang, Anhui, Fujian, Guangxi, Guangdong, Guizhou, Hainan, Henan, Yunnan, Sichuan
H4-F	KF697677, KP763784, KP763732, KP763728	Anhui, Guangxi, Hainan, Henan, Hubei, Jiangsu, Sichuan
H5-L	KF718275, KF697671, KP763803, KP763745	Guangxi, Hainan, Sichuan, Yunnan
H6-L	MG953792, KF927160, KF718269, KF697672, KP763788, KP763777, KP763761	Zhejiang, Fujian, Guangxi, Guizhou, Hainan, Henan, Yunnan, Sichuan
H7-C	MG953795, KF927155, KF697675, KP763731, KP763730, KP763729	Zhejiang, Henan, Anhui, Guangxi, Guizhou, Hubei, Jiangsu, Sichuan
H8-L*	MT816800	Sichuan
H9-L	MG953790, KF718270, KP763757, KP763746, KP763744	Zhejiang, Fujian, Guangxi, Hainan, Yunan, Sichuan
H10-L	KF718273	Hainan, Sichuan, Guangxi
H11-F*	MT816801	Sichuan
H12-L	KP763808, KP763763	Guangxi, Yunnan, Sichuan
H13-F	KP763782	Sichuan
H14-L*	MT816802	Sichuan
H15-F	MG953798, KF697676	Zhejiang, Anhui, Sichuan
H16-L	MG953799, KF718274, KP763793, KP763770, KP763749, KP763741	Zhejiang, Fujian, Guangxi, Guangdong, Hainan, Sichuan
H17-F	MG953797, KF718278	Zhejiang, Hainan, Sichuan
H18-C*	MT816803	Sichuan
H19-L*	MT816804	Sichuan

L, F, and C are the short form for 1014L, 1014F, and 1014C, respectively. The haplotypes marked with * are newly identified haplotypes in this study

### Frequency and distribution of each *VGSC* haplotype

**Fig. 4:**
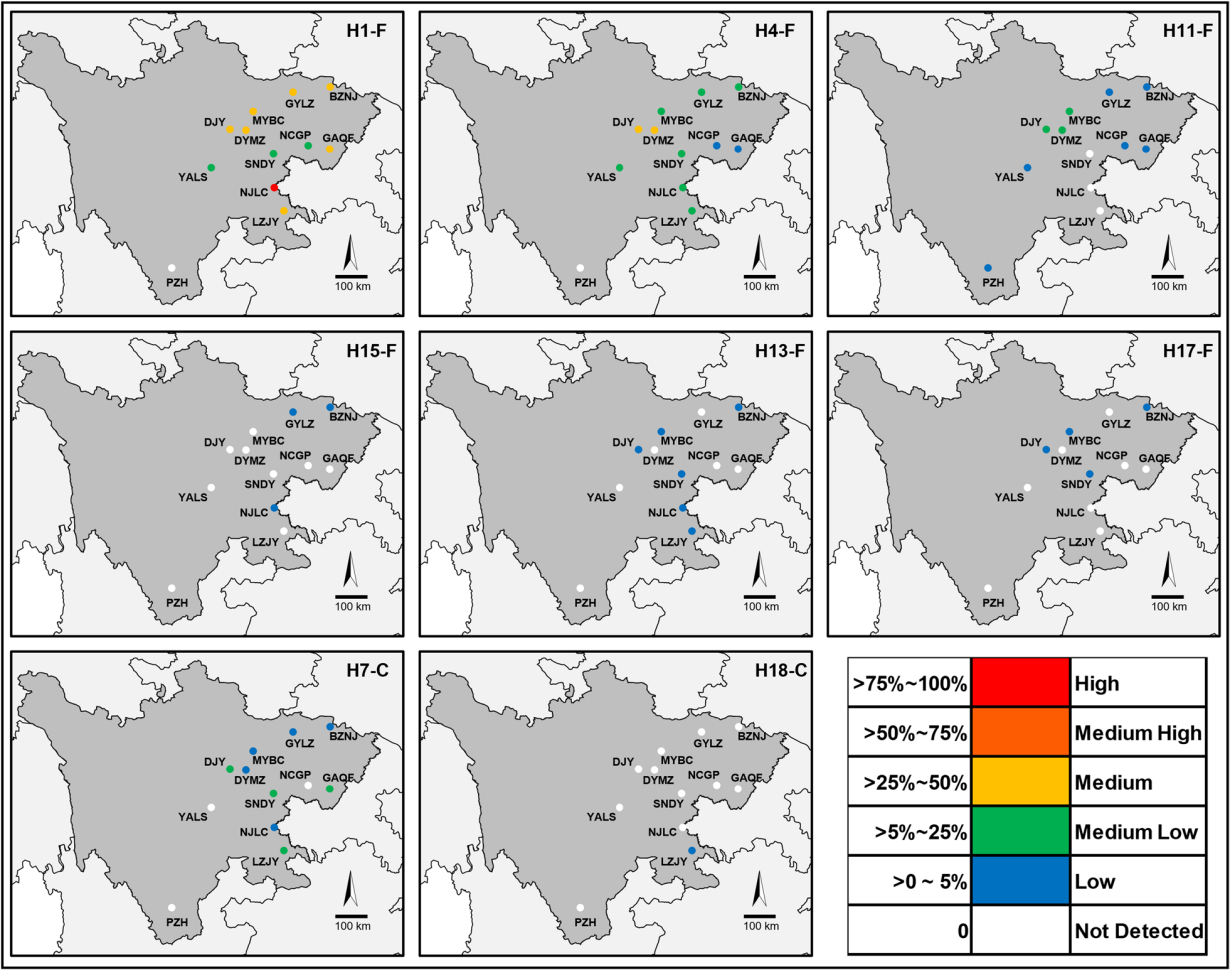
Geographic distribution of the eight *kdr* haplotypes identified in Sichuan. The frequencies of each haplotype are scaled by color based on data in Table 3

The number of haplotypes in each sampling location ranged from 6 to 13 (Table 3). H2-L and H3-L were the two common wild *VGSC* haplotypes distributed in all locations except NJLC, while H12-L and H14-L were rare and only distributed in PZH and GAQF, respectively. The overall frequency of resistance haplotypes was high in Sichuan (Table 3 and Fig. 4). Notably, H1-F was the predominant resistance haplotype across Sichuan except PZH (Fig. 4). H7-C had higher frequencies in LZJY, GAQF, and SNDY than in other sampling locations and was detected in 10 of the 12 locations, while H18-C was only distributed in LZJY at a low frequency (Table 3, Fig. 4).

**Table 3:** Geographic distribution and frequency (%) of *VGSC* haplotypes identified in this study

Haplotype	Sampling location											
	BZNJ	DJY	DYMZ	GAQF	GYLZ	LZJY	MYBC	NCGP	NJLC	PZH	SNDY	YALS
H1-F	32.1	42.1	28.2	49.7	42.5	48.8	38.8	7.5	77.2		20.5	23.1
H2-L	5.14	3.62	3.79	15.9	17.2	1.65	5.14	11.4		18.1	12.9	9.92
H3-L	2.06		8.54	5.39	6.19	3.4	6.15	33.1		29.9	8.33	21.2
H4-F	12.1	26.4	25.8	1.93	17.6	15.2	17.6	2.36	10.2		15.9	11.2
H5-L	10.1	2.04	3.47			7.41	2.48	5.96	7.39	15.8		8.22
H6-L	10.1		3.6	6.21	3.27		7.9	6.4		13.4	13.1	6.67
H7-C	4.94	6.63	2.89	10.1	1.85	13.9	2.57		1.96		11.9	
H8-L			2.25					4.41		11.6		3.57
H9-L	8.85		1.93	5.74	1.95			24			8.16	9.61
H10-L		3.37	7.96	1.54	1.79	6.14	5.05	2.68		3.55	3.92	1.86
H11-F	2.47	9.35	11.6	1.4	3.62		7.16	2.24		3.67		4.65
H12-L										3.91		
H13-F	3.91	3.25				1.84	1.29		1.56		2.03	
H14-L				2.19								
H15-F	3.09				1.43				1.68			
H16-L	2.47				2.65		2.2					
H17-F	2.68	3.2					1.93				1.64	
H18-C						1.62						
H19-L							1.75				1.81	
Number of haplotypes	13	9	11	10	11	9	13	10	6	8	11	10
Sample size	38	40	39	39	36	39	40	30	39	39	36	31

### Phylogenetic analysis of *VGSC* haplotypes

**Fig. 5:**
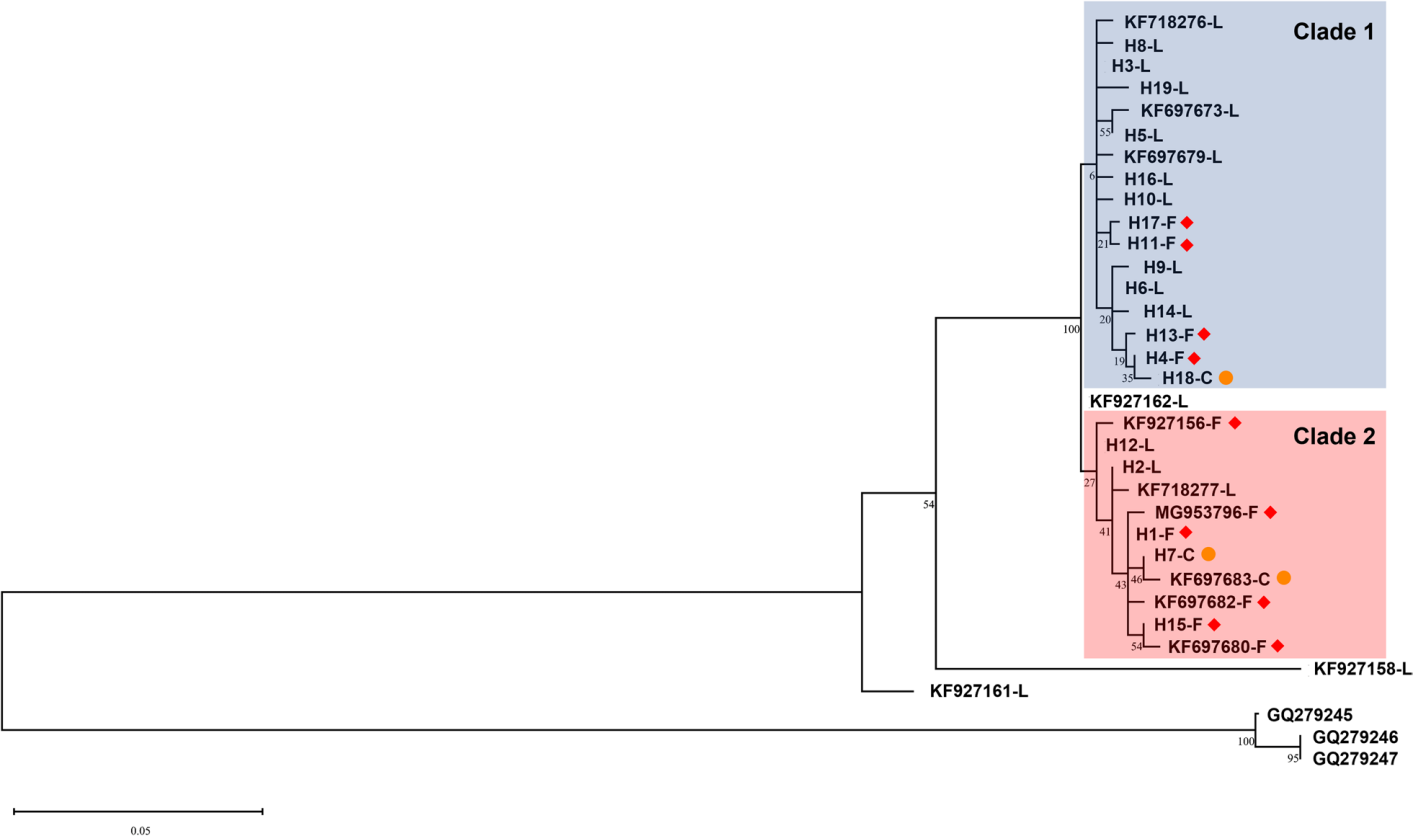
Maximum likelihood phylogenetic tree of *Anopheles sinensis* *VGSC* haplotypes. The red diamond and yellow dot represent the 1014F and 1014C haplotypes, respectively. Numbers at nodes represent the bootstrap values (%). Three *Anopheles culicifacies* *VGSC* alleles (accession numbers GQ279245, GQ279246 and GQ279247) are used as the outgroup

**Fig. 6:**
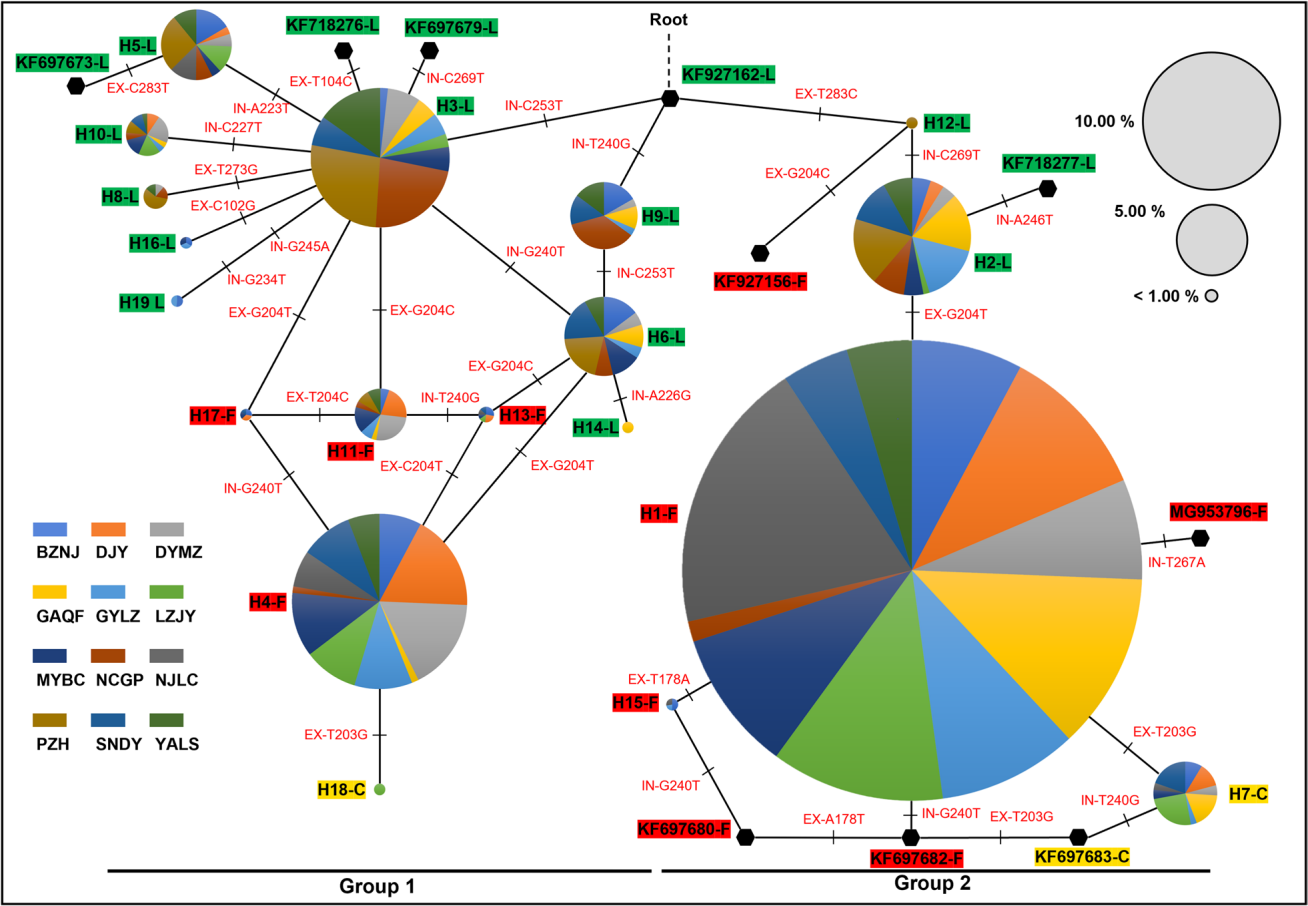
Median-joining network of *VGSC* haplotypes. Pie charts represent haplotypes detected in this study with size proportional to their frequency. Geographic regions are colored in the pie chart, respectively. Black hexagons represent the haplotype retrieved from GenBank (Table 2). Haplotypes are connected to one another based on their similarity. Different haplotypes are highlighted by colors (1014L is green, 1014F is red, and 1014C is yellow). Short hash marks perpendicular to branches indicate the number of base pair mutations between haplotypes. The character next to the short hash mark shows the mutation pattern. EX and IN are abbreviations for exons and introns, respectively

The maximum likelihood tree (Fig. 5) revealed that *VGSC* haplotypes could be clustered into two clades and both clades contained resistance haplotypes. Median-joining (MJ) network analysis (Fig. 6) showed that the 19 haplotypes identified in Sichuan could be divided into two groups. Group 1 included 14 haplotypes (H3-L, H4-F, H5-L, H6-L, H8-L, H9-L, H10-L, H11-F, H13-F, H14-L, H16-L, H17-F, H18-C, and H19-L), forming a complex reticulate network. H3-L took a center position in Group 1 and could be evolved to seven haplotypes (H5-L, H6-L, H8-L, H10-L, H16-L, H11-F, and H17-F) by one mutational step. H18-C was located at the far end of Group 1 and could be produced by one mutational step from the resistance haplotype H4-F (Fig. 6). Group 2 included five haplotypes (H2L, H12L, H1F, H15F, and H7C) with a simple evolutionary relationship. The resistance haplotype H1-F could be derived from the wild H2-L and produce two other resistance haplotypes (H15-F or H7-C) by one mutational step (Fig. 6).

## Discussion

**Fig. 7:**
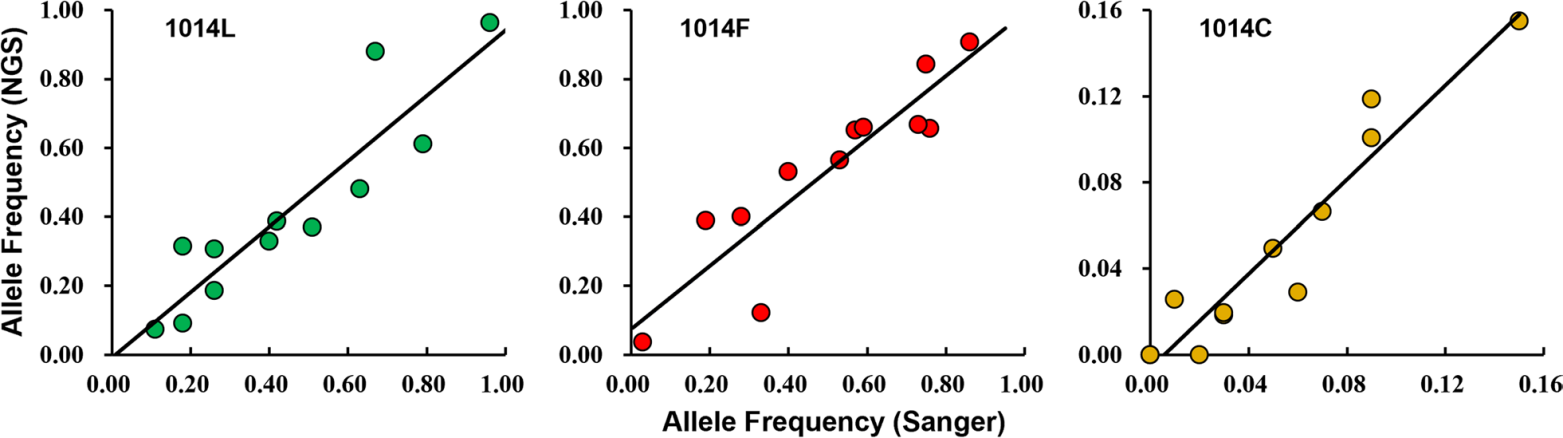
Pearson’s correlation of frequencies of *A. sinensis VGSC* alleles (1014L, 1014F, 1014C) estimated from data by Sanger sequencing (from Qian et al. 2021) and by NGS-based amplicon sequencing (this study) in 12 sampling locations in Sichuan. For 1014L (in green): *r* = 0.912 and *p*-value = 3.54 × 10^–5^; for 1014F (in red): *r* = 0.906, *p*-value = 4.84 × 10^–5^; for 1014C (in yellow): *r* = 0.954, *p*-value = 1.49 × 10^–6^

In this study, the NGS platform was deployed to understand the type and frequency of *kdr* mutation at the haplotype level. Correlation analysis showed that the frequency of *kdr* alleles detected by the NGS-based method herein was in good agreement with that determined by the Sanger sequencing [14]. Pearson’s correlation coefficients, i.e. 0.912 (*p*-value = 3.54 × 10^–5^), 0.906 (*p*-value = 4.84 × 10^–5^), and 0.954 (*p*-value = 1.49 × 10^–6^) for the frequency of 1014L, 1014F and 1014C, respectively (Fig. 7), suggest that the NGS-based method is reliable. By comparison, NGS-based amplicon sequencing is more cost-effective than Sanger sequencing [27] and can clarify other nucleotide variations in the sequence of interest for haplotype identification.

Using this NGS approach, 19 *VGSC* haplotypes of *A. sinensis* were identified in Sichuan. The eight resistance haplotypes display a patchy distribution, and each sampling location has at least one resistance haplotype (Fig. 4). Notably, H1-F is distributed in all sampling locations except PZH and has an extremely high frequency (77.2%) in NJLC, and it also is the predominant resistance haplotype in other locations (Table 3 and Fig. 4). This observation led us to postulate that H1-F may have better protection and/or lower fitness cost than other *kdr*-type haplotypes in Sichuan. Special attention thus should be paid to this haplotype in future surveillance.

Interestingly, beyond Sichuan, both H1-F and H7-C have been previously documented in Zhejiang, Henan, Anhui, Guangxi, Guizhou, Hubei, and Jiangsu provinces of China [20, 23, 28–30] (see Fig. 1 for location). The wide distribution of H1-F and H7-C in locations that are hundreds of miles apart may suggest possible gene flows between these *A. sinensis* populations, though we could not exclude the possibility of independent origins of the same haplotype in different regions.

The topology of the ML tree (Fig. 5) and MJ network (Fig. 6) strongly suggests that the resistance mutations are not singly originated. The haplotypes carrying a *kdr* mutation can be convincingly clustered into two major clades or groups. Specifically, the complex reticulate network in Group 1 supports that divergent evolution may have occurred first, followed by diversification of 1014F haplotypes driven by selection pressure (H3-L → H11-F/H17-F; H6-L → H4-F/H13-F), and eventually converged toward H4-F. By comparison, the evolutionary relationships for haplotypes in Group 2 are relatively straightforward and support multiple 1014F haplotype origins (H12-L → KF927156-F; H2-L → H1-F). Multiple origins of *kdr* mutations have also been documented in other insect species such as the house fly *Musca domestica* [31].

Multiple *kdr* mutations at position 1014 of the VGSC have been reported in several insects [32]*.* In *A. sinensis*, three types of *kdr* mutations (L1014F/C/S) have been documented [14, 23, 30]. The same mutations have been detected in a series of other anophelines [33, 34]. After reviewing the available literature, we found that the classical L1014F appears to be the first selected *kdr* allele, probably a consequence of DDT application. With global use of pyrethroids, new alternative mutations may emerge [9]. Although the level of pyrethroid resistance conferred by L1014F and L1014H replacement in the house fly is clear [35], to our knowledge, there are no published data about the effect caused by different mutations at position 1014 on insecticide resistance in *A. sinensis*. Knowledge about insecticide tolerance and fitness associated with different resistance allele will be of importance to understand the evolution of *kdr* mutations and helpful for developing pest control strategies.

Our network analysis indicated that only one mutational step is able to change TTT-encoding 1014F to 1014C. This observation confirms a sequential evolution from 1014F to 1014C in *A. sinensis* as proposed in our previous paper [23]. The codon usage may explain this notion: one mutational step can lead to the L1014F (TTG to TTT) or F1014C (TTT to TGT) substitution, while it requires two base mutations to realize the L1014C (TTG to TGT) replacement. However, this is not the case for the two resistance mutations (L1014F and L1014H) in house flies: the 1014F (TTT) allele could not evolve into the 1014H (CAT) allele (or vice versa) [9], because it would require two nucleotide substitutions.

## Conclusions

This study adopted the NGS platform for cost-effectively clarifying *VGSC* haplotypes of *A. sinensis* and provided information about the types and distribution of the *kdr*-type mutations at the haplotype level in Sichuan, China. Our results support multiple origins of *kdr* alleles and confirm a scenario of sequential evolution from TTT-encoding 1014F to 1014C in *A. sinensis*. These findings will advance our understanding about the evolutionary history of *VGSC* resistance haplotypes in the malaria vector *A. sinensis.*

## Data Availability

All datasets are presented in this article.

## Abbreviations

VGSC: Voltage-gated sodium channel
*kdr*: Knock-down resistance
NGS: Next-generation sequencing
WHO: World Health Organization
IRS: Indoor residual spray
ITNs: Insecticide-treated bed nets
ML: Maximum likelihood
MJ: Median joining
